# Pepscan Approach for the Identification of Protein–Protein Interfaces: Lessons from Experiment

**DOI:** 10.3390/biom11060772

**Published:** 2021-05-21

**Authors:** Angelita Rebollo, Eric Savier, Pierre Tuffery

**Affiliations:** 1 UTCBS, Inserm 1265, Faculté de Pharmacie, Université de Paris, 75013 Paris, France; angelita.rebollo@parisdescartes.fr; 2 Department of Hepatobiliary and Liver Transplantation Surgery, Pitié–Salpêtrière Hospital, AP-HP, Sorbonne Université, 75013 Paris, France; eric.savier@aphp.fr; 3 BFA, CNRS, UMR 8251, Inserm U1133, Université de Paris, F-75013 Paris, France

**Keywords:** PEP-scan, protein–protein interaction, interfering peptide, binding specificity

## Abstract

PEPscan is an old approach that has recently gained renewed interest for the identification of interfering peptides (IPs), i.e., peptides able to interfere with protein–protein interactions (PPIs). Its principle is to slice a protein sequence as a series of short overlapping peptides that are synthesized on a peptide array and tested for their ability to bind a partner, with positive spots corresponding to candidate IPs. PEPscan has been applied with a rather large success in various contexts, but the structural determinants underlying this success remain obscure. Here, we analyze the results of 14 PEPscan experiments, and confront the in vitro results with the available structural information. PEPscan identifies candidate IPs in limited numbers that in all cases correspond to solvent-accessible regions of the structures, their location at the protein–protein interface remaining to be further demonstrated. A strong point of PEPscan seems to be its ability to identify specific IPs. IPs identified from the same protein differ depending on the target PPI, and correspond to patches not frequently involved in the interactions seen in the 3D structures available. Overall, PEPscan seems to provide a cheap and rapid manner to identify candidate IPs, that also comes with room for improvement.

## 1. Introduction

Peptide arrays are a technology with a wide range of applications in basic and applied research, which is now nearly 40 years old and has been commercially available for about ten years. An array comprises hundreds to thousands of different peptides sequences immobilized in a solid support that can be tested simultaneously, offering many possibilities to analyse different signalling pathways between normal and pathological conditions, drug discovery, sequence dependent reactivity etc. Among the applications, one of the most common has been epitope mapping for antibodies [1], including the identification of linear epitopes between the IL-10 and its receptor [2], viral antigen epitopes [3,4], T and B cell epitopes [5]. Other applications relate to the identification of binding sequences between protein/peptide or protein/protein, screen of enzyme substrate and identification of peptides implicated in cell adhesion. These include, to cite some, the identification of novel cell adhesion peptides using a SPOT array of fibronectin peptides [6], the identification of peptides adhering to tumoral breast cancer cell line MDAMB 435 and MCF7 [7], the screening of antimicrobial peptides [8], the identification of immunogenic epitopes in individuals exposed to malaria infection [9], the detection of HLA alloantibodies in organ transplantation [10], the identification and characterization of LIR motifs on ATG8 proteins, which are small ubiquitin-like proteins critically involved in autophagy [11]. 

The usage of peptide arrays differs depending on the choice of peptide synthesis, solid support, immobilization method, size of the peptides, overlapping size of the peptide, density of spots in the membrane and detection method. SPOT synthesis is the technique the most widely used to generate peptide arrays [1,12,13,14]. It is based on solid-phase Fmoc chemistry to synthesize peptides on a membrane support. The membrane support used for arrays is usually cellulose, which is compatible with Fmoc-based peptide synthesis and the biological assay [1,15]. For other chemistries non-compatible with nitrocellulose, alternative material may be used, such as polytetrafluoroethylene (PTFE) membranes or polyethylene films [16,17,18], glass [19] or metal films. The choice of the support conditions the immobilization strategies, peptide density and detection method to analyze the array.

Although recent publications have reported labeling-free methods to analyze the arrays, such as surface plasmon resonance and mass spectrometry [20], the majority of the peptide arrays are analyzed using labeling-dependent assays. The most commonly used detection methods are based on radioactivity, chemiluminescence, colorimetry and fluorescence, the latter three being the preferred methods when using antibodies to analyze the experiment. These require an incubation with a primary antibody, that binds to the reacted peptides. The array is incubated with a secondary antibody that is usually conjugated to a reporter enzyme [21,22,23,24,25]. The membrane is then analyzed in terms of the spots associated with some signal. Of note, these assays are rapid but usually reported as giving rise to false positive results.

One method derived specifically to identify an active region of a sequence of interest is the PEPscan approach [3]. Its principle is to systematically slice a protein or a protein fragment as a series of overlapping fragments of fixed size, and to test each fragment for its binding with a partner protein using a peptide array. The most commonly used size of peptide for PEPscan ranges from 4 to 20 amino acids, with an overlap ranging from 1 to 10 peptides, while the density of the spot in a membrane ranges from a few to thousands of peptides. This to not to mention that each investigator may adapt this approach to its own needs. Although possible, the mapping of the discontinuous binding sites using PEPscan may sometimes turn more difficult due to the lower affinities of the individual fragments with the corresponding partner [26]. One of the first applications of PEPscan was the identification of epitopes recognized by monoclonal antibodies [27] as well as the recognition of T and B cells epitopes [5,28,29,30]. The epitopes recognized were mapped using a series of 15 mer peptides synthesized using the SPOT method and with a shift of five amino acids between two peptides. Finally, the PEPscan approach has recently re-gained interest due to the difficulties of designing small compounds targeting protein–protein interfaces (PPIs) [31]. According to its design, the goal of PEPscan is, in this context, to identify linear fragments corresponding to continuous sequences located at the protein–protein interface that are expected to interfere with the formation of the complex, thus affecting some biological pathway. However, although PEPscan has led to the successful identification of candidate interfering peptides (IP) showing effective biological activity, one should keep in mind that PEPscan is only an in vitro approach to identify fragments of a protein able to bind a partner it is known to bind to. The binding of a fragment in isolation might differ from the binding of the same fragment in the complete protein. The binding to the partner could trigger conformational changes, as observed for a few cases where the structure of the partner in isolation or in interaction with a peptide is known [32], biasing further interpretation. In terms of biological activity, effects upon binding could result from the binding to a region external to the PPI interface, as is the case for allosteric modulators [33], or simply come from the binding of the IP to other targets. So far, very few results have been reported about PEPscan’s ability to truly identify peptides at the protein–protein interface or its ability to identify peptides specifically interfering with a target interaction. In this study, we re-analyze the results obtained for a series of eight protein/protein interactions for which IPs were identified by PEPscan, and we compare it to the structural data available.

## 2. Materials and Methods

### 2.1. Data

**Figure 1:**
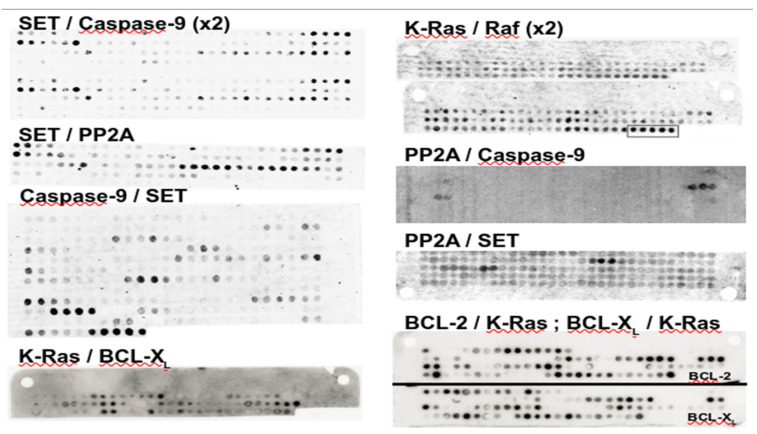
PEPscan membranes. For several cases, the images show the membranes resulting from the PEPscan experiment. Each spot in a raw image corresponds to a peptide of 12 amino acids, and consecutive spots share 10 amino acids in common. Black spots correspond to positive reacting spots. P1/P2 (e.g., SET/PP2A) indicates that P1 (resp. SET) is on the membrane that is hybridized using P2 (resp. PP2A).

Given a target PPI between two proteins, two PEPscan experiments can be undertaken: either searching for partner 1 fragments able to bind partner 2, or scanning partner 2 fragments able to bind partner 1. In the former case, partner 1 is scanned as a series of peptides—on the membrane—whose ability to bind partner 2 is tested, while in the latter case, peptides on the membrane correspond to partner 2, and their ability to bind partner 1 one is tested. In the following, we note P1/P2 in an experiment where P1 is on the membrane that is hybridized using P2. In this study, we re-analyze the results of 14 PEPscan experiments targeting 8 PPIs for which structural data is available, at least for the two partners in isolation or in interaction. Table 1 reports the UniProt [34] and Protein Data Bank (PDB) [35] identifiers of the proteins involved in each PPI, whereas Table 2 summarizes the results of each PEPscan experiment related to the PPIs. These correspond to (1) the interaction between the kinase Raf and the pro-apoptotic proteins BLC2 and BCL-X_L_ (unpublished results), (2) the phosphatase PP2A and the cysteine protease caspase-9, which is deregulated in apoptosis and tumoral transformation [36,37], (3) the interaction between the oncoprotein Ras and the kinase Raf, also deregulated in many type of cancers [38,39], (4) the interaction between the transcription factors TEAD/YAP and TEAD/Taz, implicated in the Hippo signaling pathway, also deregulated in some type of cancers, such as breast cancer and uveal melanoma [40], (5) the interaction between the phosphatase PP2A and its physiological inhibitor, the oncoprotein SET, strongly deregulated in hematological malignancies [41,42] and (6) the interaction between the cysteine protease caspase-9 and the oncoprotein SET (patent WO2016156536). For BCL-2–K-Ras and TEAD-TAZ, the results for only one PEPscan experiment out of 2 were available. Apart from the interaction between kinase Raf and the pro-apoptotic proteins BLC2 and BCL-X_L_, for which the membranes are presented in Figure 1, the raw data correspond to those of the corresponding references.

Other PEPscan experiments for which no experimental 3D structure could be identified were not included in this data set. This is the case of the experiment targeting the interaction between two proteins of the parasite *Plasmodium falciparum*, the phosphatase PP1 and the protein LRR1, involved in malaria parasite development [43], and of the recently published study targeting the phosphatase PP1 and the kinase LRRK2 involved in Parkinson disease [44] (Patent PCT 20141031).

### 2.2. PEPscan Protocol 

All the PEPscan experiments were undertaken using a constant protocol.

#### 2.2.1. Binding Assay on Cellulose-Bound Peptides 

Overlapping dodecapeptides with two amino acid shifts, spanning the complete sequence of one protein, were prepared by automated spot synthesis (Abimed, Langerfeld, Germany) on an amino-derived cellulose membrane, as described [1,12]. The membrane was saturated using 3% non-fat dry milk/3% bovine serum albumin (BSA) (2 h, room temperature) incubated with purified partner protein (overnight, 4 ℃) and after several washing steps, incubated with an antibody against the protein partner (2 h, room temperature) followed by horse radish peroxidase (HRP)-conjugated secondary antibody (1 h, room temperature). Positive spots were visualized using the ECL system (Bio-Rad).

#### 2.2.2. Peptide Synthesis and Sequence 

Peptides were synthesized in an automated multiple peptide synthesis with a solid-phase procedure and standard Fmoc chemistry. The purity and composition of the peptides were confirmed by reverse-phase high-performance liquid chromatography (HPLC) and mass spectrometry.

### 2.3. Candidate Peptide Identification from Membranes 

For the analysis of the PEPscan membranes, a specific question arises from the partial redundancy of the consecutive spots that share the overlapping residues. Commonly noticed is that there is no standard number of consecutive reacting spots revealing a positive interaction. In general, it is accepted that a positive binding site requires the presence of at least 3 consecutive positive spots with a strong enough signal, sometimes with a difference in the intensity signal between the medium spot and the end spots. However, this is not a general rule, as illustrated in Figure 1. Occasionally, a ringspot effect is observed. This corresponds to spots for which a stronger signal of hybridization is observed on the ring of the spot compared to the center of the spot. This irregular signal can make the choice between binding and non-binding [45]. One possible explanation for this effect is a possibly higher concentration of the peptide in the center of the spot, resulting in the exhaustion of the signal before detection. Another explanation is a concentration gradient of the amino acids from the center to the ring of the spot during synthesis, giving a lower concentration of peptide in the core of the spot. These spots are not considered in a particular manner in our study. Here, we have considered the IPs reported by the authors of the studies, but our re-analysis of the membrane sometimes led us to accept newcomers to better sense the significance of the positives in terms of 3D structure (see Table 1).

Since the sequences of the consecutive spots overlap here by all but two, i.e., by 10 amino acids, the spots of a series of consecutive positive spots usually share only a few amino acids. For instance, for a series of 3 consecutive positive spots, 16 amino acids are involved, but the common part of the 3 sequences correspond to the central 8 amino acids. The fragments in common in the largest number of spots, are here referred to as Maximally Overlapping Fragments (MOFs), and the sequences resulting from the union of all the spots are here referred to as candidate IPs.

### 2.4. Structurally Validated Patches of Interaction 

To analyze the specificity of the binding patches, we have searched for structures containing several chains including the proteins of Table 1. For each sequence, we have used blast [46] against the sequences of the PDB with an e-value < 10^−10^ to identify close homologs of the proteins. We have then analyzed which PDB entries encompass several chains (i.e., correspond to complex structures) and identified the chains in interaction with the query. Not to bias the results, we have discarded the interologs (complexes made of homologs) for which each chain has over 70% sequence identity with a previously identified complex. Doing so, we expect to obtain information about only the close homologs of the query, i.e., members of the same family or very close families, and obtain information on how specific the interaction of the associated peptide with a particular target could be, regarding the other structurally characterized interactions of this target, accepting close homologs should interact similarly. For instance, for PP2A, this procedure identifies complexes involving not only the PP2A catalytic chain, but also other phosphatases, such as PP1, PP5, etc. The propensity of a residue to be located at an interface was then simply set up as the number of times it was at an interface divided by the number of complexes identified. Z-scores were calculated on a per protein basis, using as reference the mean and standard deviations of the propensities over the whole ensemble of residues of the protein, and as observation the propensities averaged over the residues of the IPs. Positive values indicate a tendency to be located more frequently at PPI interface, while negative values indicate a tendency to be located less frequently at PPI interface.

## 3. Results 

### 3.1. A Note on PEPscan Experiment Reproducibility 

It is important to note that the relationship between the signal intensity and the affinity of the peptide/ligand interaction is not straightforward. Several factors may influence the correlation between signal intensity and binding affinity; the synthesis can lead to different amounts of peptide in the spot and the type of detection assay used can also have an influence. In addition, the non-homogeneity of the cellulose membrane can influence the results [45,47]. In general, it is accepted that this type of approach is not well suited to quantify the interaction affinity. Importantly, however, peptide array experiments are fairly reproducible, as illustrated in Figure 1, which presents duplicates of one experiment. Some variations in the intensities of the spots can be observed, but strong signals appear for equivalent spots, meaning that usually, only one experiment is enough to obtain the information that is sought after.

### 3.2. Confronting PEPscan Positive Fragments with Structural Data 

Successes achieved using PEPscan for the identification of protein fragments able to interfere with protein–protein interactions raise numerous questions in terms of structure. First, the conformation adopted by the peptides synthesized using SPOT technology is largely unknown and, particularly, the impact of the linker binding to the support (cellulose or other). Second, the impact of splitting proteins into fragments on their conformational balance is also unknown. Third, the strategy to systematically consider all possible fragments, which is necessary when not knowing/considering the structure, also comes with numerous questions related to the behavior of the core hydrophobic fragments in terms of possibly non-specific binders. Finally, mechanisms for peptide interference with protein–protein interactions could also reveal greater complexity than the straight disruption of the 3D interaction patch. We now discuss these aspects from a series of results on interfering peptide identification.

**Figure 2:**
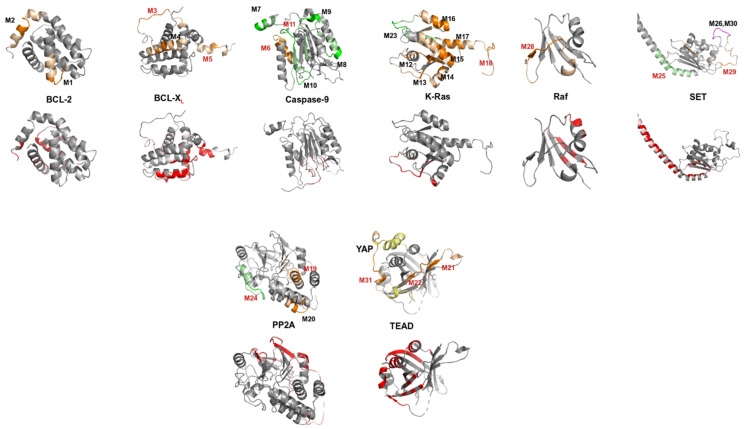
Top: For each of the 8 proteins having undergone a PEPscan, the location of the IPs in the structure is presented. For cases where the IPs of different PEPscans are presented, different colors are employed (green vs. orange). Darker shades correspond to the MOFs. MOFs are labeled as in Table 2. Red labels correspond to IPs showing some biological activity. Bottom: the residues most interacting in the complexes of structure available in the PDB are depicted using a red color gradient.

Table 2 presents all the candidate IPs for 14 PEPscan experiments. The MOFs are in bold. Details on the secondary structure of the fragments corresponding to the IPs show a diversity of topologies. Figure 2 shows, when possible, the location in the 3D structure of the IPs and the MOFs.

### 3.3. PEPscan Identifies a Limited Number of IPs 

A first observation is that overall, one denotes the existence of a specific answer depending on the PPI studied. Indeed, the IPs identified for the same protein with two different partners are all different, with the exception of SET, for which M26 and M30 have a large overlap. A second observation is that the number of candidate IPs is, as expected, fully unrelated to the size of the protein and, for most of the cases, it is rather low (2.2 on average). It is more than four for only two cases. One such example corresponds to the K-Ras/Raf interaction, for which many spots appear positive, and one region (C-terminus) shows a stronger signal in experiment 2 (Figure 1). The second one corresponds to the caspase-9/SET interaction for which several series of positive spots are lighter than others (Figure 1). For this case, one observes some heterogeneity in the intensities of the spots, and high-intensity spots are observed for only four series. Overall, the very low number of IPs per PEPscan experiment would support PEPscan IPs’ suitability for systematic testing downstream. A final remark is that the only IP detected that corresponds to only two consecutive positive spots is M20, but it shows no activity, which is in agreement with the rule that IP detection should involve at least three consecutive spots.

### 3.4. Can MOFs Correspond to Fragments at the PPI Interface? 

We now turn to considering whether the IPs can target PPI interfaces. As shown in Figure 2, the MOFs correspond to regions of the structures that are exposed to the solvent, which fulfils a requirement for the identification of motifs at the interface of protein complexes. Looking in more detail at if the MOFs could correspond to fragments at the interface of PPIs is limited by the number of cases for which the structure describing the PPI is available, which is often the rule in the context of PEPscan experiments. In our dataset, the structures of the proteins in interaction are only available for K-Ras/Raf, TEAD/YAP and TEAD/Taz, and the PDB identifiers are 4g0n, 3kys and 5gn0, respectively. The release dates of the 3kys and 5gn0 PDB entries are 2010 and 2017, i.e., synchronous or posterior to the publication of the PEPscan results for TEAD/YAP/Taz (2010).

For the K-Ras/Raf interaction, one MOF (M13) is clearly located at the complex interface. Interestingly, however, M13 does not correspond to the IP showing some activity. In fact, it was not originally considered as a candidate by the experimenters and was consequently not tested. M18, which is not close to the interface, was instead tested and shows some activity, including in terms of competition, meaning it is able to interfere with the formation of the K-Ras/Raf complex [39]. It is important to recall, however, that the structure of the complex does not encompass the totality of the sequences, suggesting alternative mechanisms underlying the interaction could occur. On the Raf side, none of the IPs are located at the interface of the K-Ras/Raf complex.

For the TEAD/YAP and TEAD/Taz interactions, two IPs, M22 and M3,1 are clearly located at PPI interaction sites in the 3D structures, which is not the case with M21, which is located at the opposite side of the structure (Figure 2). M22 is surprising, since it corresponds to a beta strand internal to a seven-stranded beta-sheet, but an interaction can clearly occur, particularly through TRP 303, which belongs to the associated MOF. For M31, the MOF is close to, but not in direct interaction with, that of M22. In terms of activity, it is again intriguing that the competition experiments [40] suggest that M21 is able to disrupt the TEAD/YAP complex. However, in this case again, the coordinates are only available for a linker part of the YAP and TAZ paralogous proteins, meaning other mechanisms of interaction not covered by the resolved part of the structure could be at stake. Moreover, the ELM repository [49] also suggests that some interactions would occur between TEAD and YAP through short linear motifs, suggesting the cellular effects observed could involve mechanisms not necessarily specific to the TEAD/YAP interaction.

Overall, despite that these results tend to suggest that PEPscan is able to identify, with some fuzziness, peptides at the PPI interface, the cellular tests highlight that the biological reality could be much less straightforward.

### 3.5. Can Candidate IPs Target Specific Patches on the Partner Protein?

**Figure 3:**
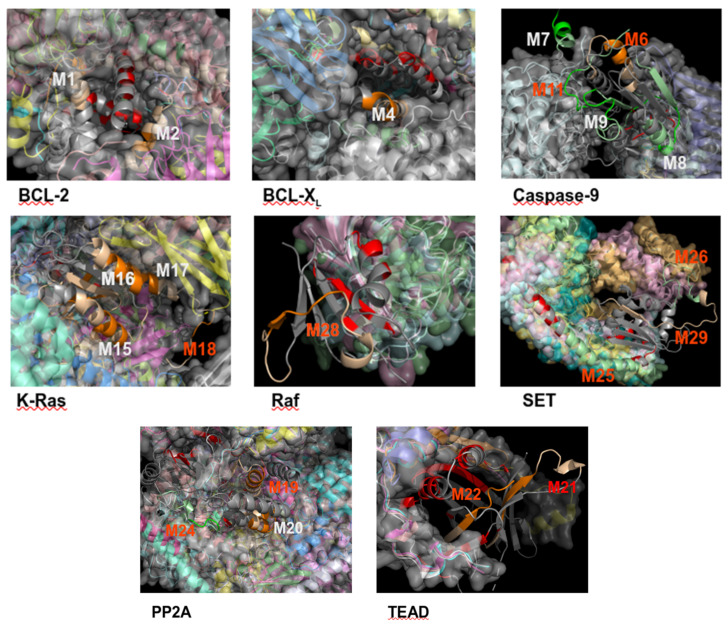
3D illustration of the surroundings of the IPs when not frequently located at the protein–protein interface. Red labels correspond to IPs showing some biological activity.

A last aspect is the specificity of the putative interaction sites identified, and firstly the ability to identify IPs specific to a given interaction. In our dataset, IPs extracted from the same protein but targeting a different PPI are available in the cases of caspase-9 (caspase-9-SET, caspase-9-PP2A), PP2A (PP2A-caspase-9, PP2A-SET), SET (SET-PP2A, SET-caspase-9) and K-Ras (K-Ras-BCL-X_L_ and K-Ras-Raf), not considering the special case of TEAD. In Figure 2 (top), different color codes are used to depict the IPs identified on one protein, but targeting different PPIs. For all cases but one, the IPs identified involve different regions of the structures. This is particularly obvious for caspase-9, PP2A and K-Ras. SET is the only case where some IPs targeting PP2A or caspase-9 correspond to overlapping sequences. M26 and M29 can be considered as consecutive, and overlap by only four amino acids (169-RSSQ). M26 and M30 are almost fully overlapping. Here, we depict a full model where missing regions have been added by DaReUS-Loop [50,51], but these IPs correspond to parts of the structure predicted as disordered, and for which the coordinates are missing in PDB entry 2E50. It remains that all the IPs showing some biological activity (labeled in red in Figure 2 and Figure 3) are fully distinct over all the cases analyzed.

Another aspect is the fact that the IPs could be non-specific in terms of interference, i.e., able to target different PPIs. To investigate this, we have analyzed the structural information available in the PDB about the interactions of each of the proteins of Table 1. Figure 2 (bottom) presents an overview of the regions contacted by the members of the complexes identified (the redder, the more amino acids are at the interface with other protein chains), using the same orientation as for the MOFs (top). Figure 3 provides details on the 3D environment of the less-contacted MOFs. MOFs that correspond to peptides showing some activity (Table 2) are labeled in red. Table 2 also reports z-scores as a quantification of the tendency of the IPs to be located more (positive values) or less (negative values) frequently at the PPI interface than the rest of the amino acids.

It is striking that the MOFs are not, in general, located in the structure regions frequently involved in complex interfaces, suggesting some specific mode of interaction could be grabbed. Positive z-score values are obtained only for M5 (BCL-X_L_), M10 (caspase-9), M13-14 (K-Ras) and M25 (SET). Among these, only M25 shows some activity (see Table 2). It could correspond to a peptide that could interfere with the dimerization of SET (Figure 3). For M5, we identified 27 complexes of the PDB involving BCL-X_L_ or close homologs. Among these, 24 correspond to BCL-X_L_ in interaction with peptides designed to prevent the BCL-X_L_ heterodimerization that is involved in the regulation of programmed cell death. Those peptides are extracted from various partners (BAK, Bad, Puma, Beclin, BIM) or correspond to foldamers. It is thus legitimate to expect that M5 can be involved in the interaction with K-Ras, but the specificity of its interaction could probably be questioned. Overall, among the nine MOFs that exhibit some activity and for which 3D coordinates are available, all but one (i.e., eight IPs) have negative z-scores, i.e., correspond to regions less frequently contacted by partners (Figure 3), and thus could be considered as potentially specific to the interaction they target. Of course, this has to be tempered by the possible lack of structure in the PDB, and z-score values here are only indicative. A special case is that of M22 (TEAD), associated with a z-score of -0.24, for which very few complex structures of the PDB could be identified (only seven). Three of these correspond to TEAD in interaction with YAP, and another one to Taz. Others describe the interaction with VGLL. Hence, M22 can be expected to correspond to a true positive case, but clearly, the specificity cannot be assessed.

## 4. Discussion

PEPscan is an old approach that has recently raised new interest for the identification of IPs. PEPscan has been previously reported to sometimes generate false positives. The way it is employed here for IP identification, the number of hits per target appears rather limited. Indeed, only for one case (K-Ras) does the number of candidate IPs exceeds five. The average and median number of IP are respectively 2.21 and 2, which is fairly low.

Out of the 14 analyzed experiments, PEPscan was able to identify IPs showing some biological activity for all cases but two, i.e., a success rate in identifying IPs of close to 86%. The mechanisms underlying this activity are, however, less clear. If all the MOFs correspond to regions that are solvent-accessible, i.e., likely to be at the PPI interface, the analysis of the two examples for which structural information about the binding mode of the partners exists is limited in both cases by the lack of coordinates for large parts of the proteins. In some cases, IPs showing some activity are not located at the PPI interface of the regions of resolved structure. It is also noteworthy that for the K-Ras/Raf interaction, the fragment of Raf at the PPI interface did not show any signal in the PEPscan experiment. Reasons for this are unclear, and could come from the intrinsic properties of the associated sequence fragments. Looking at the aggregation propensities of the Raf sequence, various regions were predicted as aggregation-prone by the Walz server [52], but not in the region 66–71 located at the interface. It remains that PEPscan was able to identify a spot at an experimental 3D PPI interface in three cases out of five, further analysis being limited due to the absence of 3D coordinates.

A very promising aspect of this study is that our analyses suggest that when applied to IP identification, the PEPscan approach seems able to identify functional IPs that are specific to the target PPI. Indeed, functionally active IPs identified from one protein to address different PPIs correspond in all cases to different fragments of the protein. Of note, the only case of protein SET, with one IP identified for the PP2A/SET interaction, was largely overlapping with that identified for the caspase-9/SET interaction, but was not active. Conversely, for eleven out of fifteen (73%) of the functional IPs identified, the fragment corresponds to a region of the protein not frequently involved in the assemblies of 3D structure determined, available in the PDB. This last observation must be, however, taken with care, since the structural data available in the PDB are far from covering the complete set of interactions expected in a cell [53,54].

Overall, it seems obvious that SPOT peptide arrays should continue playing a key role in the above-described applications, as well as for new purposes. From our analyses, this approach combines the advantages of being rather successful while being cheap and quick to enforce. In addition, we have here analyzed the results of experiments obtained using a constant protocol, proven effective over years, but there is room for evolution concerning the solid support, the array density and the size of the immobilized peptides, as well as the size of the overlapping sequence. The object of the interactions could also vary. Presently limited to proteins, the binding of different classes of molecules could be foreseen, such as nucleic acids, lipids and small compounds, to cite some. Continuing on PPIs, many perspectives are still open, particularly for therapeutic applications. PEPscan seems of particular interest when the structure of the partners in interaction is not known, and when in silico analyses cannot reliably be conducted. This is the case in numerous pathologies, such as various cancers, for which identifying a peptide can benefit from the coupling with cell-penetrating peptides [31], and more recently from tumor-addressing peptides [55].

## Figures and Tables

**Table 1:** UniProt and PDB identifiers of the proteins.

Protein	UniProt	Structure
BCL-2	P10415	2xa0
BCL-X_L_	Q8SQ42	4qnq/1bxl
Caspase-9	P55211/Q8C3Q9	1jxq/2ar9
K-Ras	P32883-2	5uk9
PP2A	P67775	5w0w
Raf	P04049	3omv/4g0n
SET	Q01105-2	2e50
TEAD	Q15562	3kys/3l15
YAP	P46937	3kys
TAZ	Q9GZV5	5gn0

**Table 2:** IPs identified by PEPscan.

PEPscan	IPs									
	P1					P2				
	Lbl	Seq	s2	z-sc	Val.	Lbl	Seq	s2	z-sc	Val
BCL-2/K-Ras (×1)	M1	19-IHYKLSQRGYEWDAGDVG	αc	−0.33	-					-
	M2	187-TWIQDNGGWDAFVELYGP	αc	−0.14						
BCL-XL/K-Ras (×1)	M3	27-FSDVEENRTEAPEGTESE (*)	-	-	-	M23	111-MVLVGNKCDLPSRTVDTK	βc	−0.53	-
	M4	163-VSRIAAWMATYLNDHLEP	α	–0.44						
	M5	195-YGNNAAAESRKGQERFNRWFLTGM (*)	-	0.58						
Caspase-9/PP2A (×2)	M6	401-YIETLDGILEQWARSEDL (†+)	αc	−0.28	cpm, cell, aff	M24	175-DTLDHIRALDRLQEVPHEGP (†+)	αc	−0.36	cell
Caspase-9/SET (×2)	M7	133-SGGFGDVGALESLRGN (n)	α	−0.39	cell					cell
	M8	199-FMVEVKGDLTAKKMVLAL (n)	βα	−0.2						
	M9	267-FNGTSCPSLGGKPKLFFI (n)	cβ	−0.39		M25	53-ILKVEQKYNKLRQPFFQKRSEL (†+)	α	0.94	
	M10	348-FPGFVSWRDPKSGSWYVE (n)	cα	1.88		M26	169-RSSQTQNKASRKRQHEEP (†-)	cα	−0.22	
	M11	399-QMPGCFNFLRKKLFFKTS (†+)	βc	0						
K-Ras/Raf (×2)	M12	1-MTEYKLVVVGAGGVGK (n)	βc	−0.19	cpm, cell					cell
	M13	31-EYDPTIEDSYRKQVVIDG (3D site) (n)	cβ	1.74						
	M14	61-QEEYSAMRDQYMRTGEGFLCVF (n)	cαβ	0.19		M27	1-MEHIQGAWKTISNGFGLK (*†+)	-	-	
	M15	91-EDIHHYREQIKRVKDSED (n)	αc	−0.46		M28	103-HEHKGKKARLDWNTDAAS (†+)	cβc	−0.42	
	M16	127-TKQAQELARSYGIPFI (n)	αc	−0.34						
	M17	155-AFYTLVREIRKHKEKMSK (n)	α	−0.4						
	M18	169-KMSKDGKKKKKKSRTRCTVM (†+)	αc	−0.34						
PP2A / SET (×1)	M19	95-ETVTLLVALKVRYRERIT (†+)	αc	−0.28	cpm, cell	M29	151-PSSKSTEIKWKSGKDLTKRSSQ (†+)	Βcα	−0.59	cell
	M20	133-CLRKYGNANVWKYF (n)	α	−0.37		M30	165-DLTKRSSQTQNKASRKRQHEEP (n)	αcα	−0.28	
TEAD / YAP (×1)	M21	227-RLQLVEFSAFVEPPDAVD (†+)	βc	−0.52	cpm, cell	M31	76-KTANVPQTVPMRLRKLPD (†+, 3D site)	c	-	cell
TEAD / TAZ (×1)	M22	293-PPHAFFLVKFWADLNWGPSGEEAGAG (†+, 3D site)	ββ	−0.24	cell					cell

For each PEPscan experiment of identifier “protein1-protein2”, P1 (resp. P2) corresponds to a PEPscan experiment in which the membrane containing the protein1 (resp. protein2) fragments is hybridized with protein2 (resp. protein1). The number of duplicates of the experiment is indicated as x1 or x2. Lbl: the unique label of each motif (see Figure 2). Seq: the sequence of the motifs in the UniProt sequence (see Table 1). s2: schematic organization in terms of secondary structure, as determined using dssp [48]. z-sc: specificity z-score (see methods). Val.: Experiment used to probe the motif’s activity (cmp: in vitro competition, cell: physiological test, aff: binding affinity measurement, 3D site: the IP is located at PPI interface). Only the IPs labeled using (†) have been tested experimentally (see corresponding references). (†+): peptides showing some activity. (†-): peptides not showing any activity. *: IPs without coordinates in the 3D structures. (n) Peptides not previously reported, but considered during membrane re-analysis

## Data Availability

Not applicable.
